# Validation of an online questionnaire for identifying people at risk of familial and hereditary colorectal cancer

**DOI:** 10.1007/s10689-015-9792-1

**Published:** 2015-03-24

**Authors:** F. G. J. Kallenberg, J. E. G. IJspeert, P. M. M. Bossuyt, C. M. Aalfs, E. Dekker

**Affiliations:** 1 Department of Gastroenterology and Hepatology, Academic Medical Center, University of Amsterdam, Meibergdreef 9, Amsterdam, 1105 AZ The Netherlands; 2 Department of Clinical Epidemiology, Biostatistics and Bioinformatics, Academic Medical Center, University of Amsterdam, Amsterdam, The Netherlands; 3 Department of Clinical Genetics, Academic Medical Center, University of Amsterdam, Amsterdam, The Netherlands

**Keywords:** Questionnaire, Family history, Pedigree, Lynch syndrome, Familial colorectal cancer

## Abstract

We developed and validated an online questionnaire to document familial cancer history, in order to facilitate the detection of persons with a familial or hereditary colorectal cancer (CRC) risk. The development of the self-administered online questionnaire for the assessment of familial and hereditary CRC risk was based on nationwide criteria for referral to genetic specialists due to a Lynch syndrome suspicion, as well as existing criteria for surveillance colonoscopies because of an increased risk of familial CRC. The questionnaire was validated at a private colonoscopy center. Patients scheduled for colonoscopy were enrolled (n = 150). Performance of the questionnaire was assessed by comparing referrals based on questionnaire data against referral decisions based on full pedigree data. In a second validation phase, referrals based on questionnaire data were compared with referrals based on data collected in a telephone interview. We also calculated inter-observer agreement in referral decisions. In the first validation phase, the questionnaire had a sensitivity of 90 % (95 % CI 55–98 %) at a specificity of 98 % (95 % CI 87–100 %) in identifying persons qualifying for referral. In the second validation phase, sensitivity was 100 % (95 % CI 63–100) at a specificity of 97 % (95 % CI 91–99 %). In both validation phases an inter-observer agreement of 100 % in referral decisions was achieved. The online questionnaire has a high sensitivity and specificity in identifying persons qualifying for referral because of suspected Lynch syndrome or familial CRC. Implementation of this tool in colonoscopy clinics can facilitate the detection of patients with hereditary or familial CRC.

## Introduction

Colorectal cancer (CRC) is the second most prevalent type of cancer in the Netherlands with more than 13.000 newly diagnosed patients per year [1]. The lifetime risk of developing CRC in a Western population is 5–6 % [1–3]. Of all CRC cases, 15–20 % are related to familial or hereditary factors [4–6].

The most common form of inherited CRC is Lynch syndrome, which comprises 2–4 % of all CRC cases [7]. This syndrome is caused by an inherited mutation in one of the mismatch repair genes and is characterized by a predisposition to develop CRC and several extra-intestinal malignancies, such as endometrial, gastric and ovarian cancer, at a relatively young age [8, 9]. Lynch syndrome is usually suspected based on the internationally used Amsterdam I and II criteria and the Revised Bethesda criteria [10, 11]. Other hereditary types of CRC include several polyposis syndromes, such as familial adenomatous polyposis and MUTYH-associated polyposis.

In familial CRC patients no genetic mutation can be found. The definition of this syndrome is based on the number and age at diagnosis of relatives with CRC. Patients who are suspected of having an inherited CRC syndrome in whom no genetic mutation is found can also be referred to as familial CRC patients [10, 11]. These patients have a threefold or higher risk of developing CRC [10, 11].

International guidelines recommend surveillance colonoscopies for both patients with familial CRC or Lynch syndrome [10–13]. Strict surveillance can reduce morbidity and mortality from CRC by up to 80 % [13–15]. Patients with an increased CRC risk are also advised to warn their relatives, who can subsequently consult a clinical geneticist for evaluation and surveillance recommendations. Additionally, if Lynch syndrome is diagnosed in a patient with CRC, surgical treatment might be adjusted; usually a subtotal colectomy is advised instead of a partial resection [16].

Lynch syndrome and familial CRC often go unrecognized by physicians and patients [6, 17–24]. Many physicians seem to be limited in their ability to apply criteria for assessing familial risk and surveillance strategies, and they do not seem to sufficiently explore family history [17–20]. Most CRC patients and their relatives do not themselves have sufficient information and knowledge to assess their eligibility for genetic referral [21, 22]. As a consequence, only a small proportion of CRC patients and their relatives who would qualify for referral is appropriately referred to a clinical geneticist (approximately 15–30 %) [6, 17, 23, 24].

To adequately document family history and to increase the detection of persons with a risk of familial or hereditary cancer, several tools have been developed. These range from paper or digital questionnaires to structured interviews [25–29]. However, for its use in daily practice no online self-administered family history tool exists for identifying persons with an increased risk of CRC. We developed and validated a new, self-administered online questionnaire to document family history, to facilitate the detection of persons with suspected familial or hereditary CRC syndromes.

## Materials and methods

### Questionnaire development

Our questionnaire was built on the criteria for referral to a clinical geneticist or gastroenterologist, as formulated in the Dutch national guideline on hereditary CRC [11]. These criteria are based on the internationally used Amsterdam and Revised Bethesda criteria for the detection of Lynch syndrome [30–32]. Criteria for familial CRC are also formulated in the guideline, as well as criteria for a screening colonoscopy for persons, who have a CRC family history that does not meet the criteria for familial CRC or Lynch syndrome. For familial CRC as well as for patients with an indication for a single colonoscopy, a referral to a clinical geneticist is not indicated, but colonoscopy screening or surveillance recommendations are made.

**Fig. 1:**
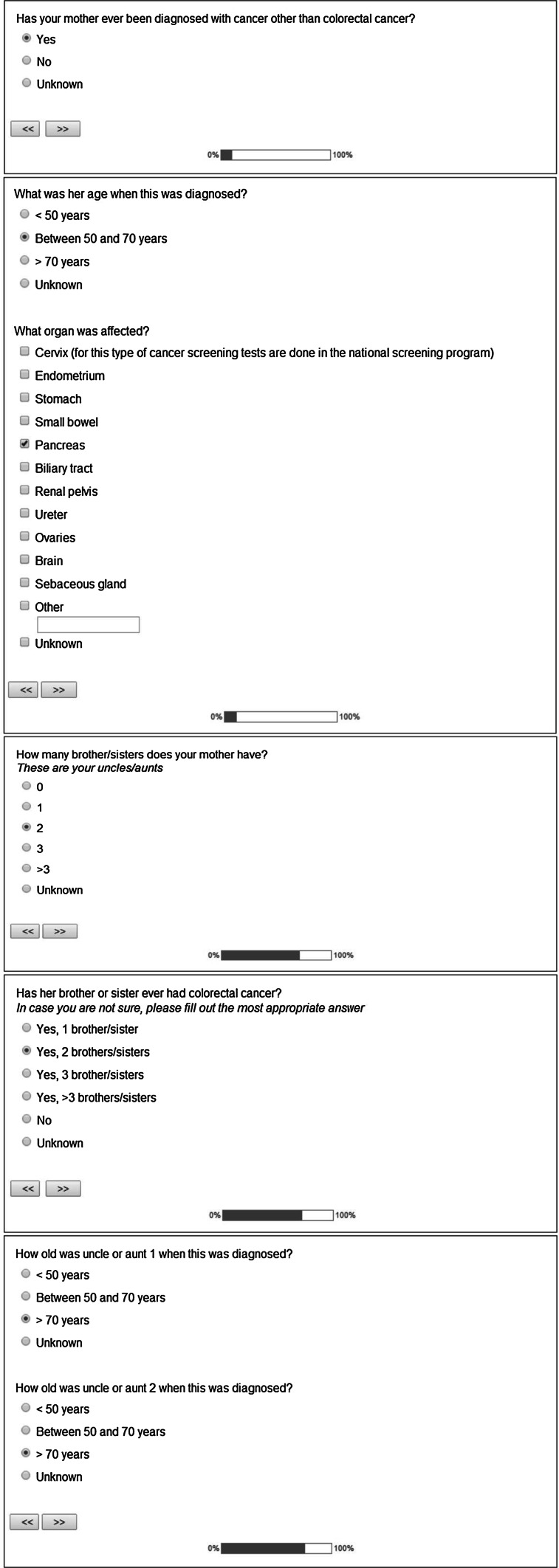
Screenshots of the questionnaire

Both the patient’s cancer history as well as the cancer history of first and second degree relatives are systematically queried in the questionnaire, by using conditional questions about affected as well as non-affected relatives. An example of the questions can be found in Fig. 1. We mainly focused on CRC and Lynch syndrome associated tumors (LSAT), but people were asked to note all cancer types that occurred in their families. Carcinoma of the endometrium, stomach, small intestines, pancreas, bile ducts, renal pelvis, ureters, ovaries, brain and carcinoma or adenoma of the sebaceous gland were considered LSAT [11].

Based on the questionnaire responses, a referral decision can be made, according to the criteria mentioned earlier. Several small adaptations were made to these criteria for making referral decisions (Tables 1, 2, 3). As the guideline referral criteria do not differentiate between maternal and paternal lineage, we only referred patients with multiple relatives with CRC or LSAT if these relatives were from the same lineage in order to increase specificity. In case a relative had a synchronous or metachronous CRC or LSAT we counted this as two relatives with CRC or LSAT, to compromise for the fact that the occurrence of a synchronous or metachronous CRC or LSAT in relatives is not incorporated in the Dutch referral criteria despite the increased risk of Lynch syndrome in these situations. We also referred patients with an LSAT and with two first or second degree relatives with CRC or LSAT, all younger than 70 years and patients with CRC younger than 70 years with a first or second degree relative with CRC younger than 70 years. We did not implement criteria that we considered too medically detailed for a patient, such as ‘having a first degree relative with a microsatellite instable CRC’. If patients had a relative with cancer diagnosed at an unknown age, we assumed an age range between 50 and 70 years, in order not to miss any patients at risk.

**Table 1:** Referral criteria for Lynch syndrome

A patient with colorectal cancer or endometrial cancer <50 years
A healthy person with a first degree relative with colorectal cancer or endometrial cancer <50 years
A healthy person with a family member with a known mismatch repair mutation
A healthy person with at least three first or second degree relatives with colorectal cancer or a Lynch syndrome associated tumor^a^, all diagnosed <70 years^b^
A colorectal cancer patient with a synchronous or metachronous colorectal cancer <70 years
A colorectal cancer patient with a synchronous or metachronous Lynch syndrome associated tumor^a^ <70 years
A colorectal cancer patient with a first degree relative with colorectal cancer or a Lynch syndrome associated tumor^a^ <50 years
A patient with colorectal cancer or a Lynch syndrome associated tumor^a^ with at least two first or second degree relatives with colorectal cancer or a Lynch syndrome associated tumor^a^, all diagnosed <70 years^b^

^a^Lynch syndrome associated tumors: carcinoma of the endometrium, stomach, small intestines, pancreas, bile ducts, renal pelvis, ureters, ovaries, brain and carcinoma or adenoma of the sebaceous gland

^b^Family members must all be genetically related (paternal or maternal lineage)

**Table 2:** Referral criteria for familial colorectal cancer

A healthy person with two first degree relatives with colorectal cancer 50–70 years^a^
A healthy person with a first degree relative with colorectal cancer 50–70 years and a second degree relative with colorectal cancer <70 years^a^
A colorectal cancer patient with a first degree relative with colorectal cancer both 50–70 years
A colorectal cancer patient 50–70 years and a second degree relative with colorectal cancer <70 years

^a^Family members must all be genetically related (paternal or maternal lineage)

**Table 3:** Referral criteria for a single colonoscopy^a^

A person with two first degree relatives with colorectal cancer >70 years^b^	

^a^If criteria are met a single colonoscopy at the age of 65 years will be offered

^b^Family members must all be genetically related (paternal or maternal lineage)

During the development phase of the questionnaire health care professionals (gastroenterologists, clinical geneticists) commented on several versions, to improve content validity. Subsequently, a preliminary version was pilot-tested on ten consenting patients. If questions were found to be unclear or irrelevant, they were removed or adapted. The revised version of the questionnaire was introduced in July 2013.

The burden for the responder was kept low by means of simple, short and distinct questions. All but one question were provided with bullet-point answers. If a cancer type was not mentioned in the bullet-point answers, an open text box could be filled out with the appropriate cancer type. The questionnaire consists of conditional questions to avoid irrelevant questions. This results in a minimum number of 13 and a maximum number of 117 questions to be answered.

### Questionnaire validation

We evaluated the validity and inter-observer agreement of the online questionnaire, using the quality criteria of the Scientific Advisory Committee of the Medical Outcomes Trust [33]. This evaluation was conducted between July 2013 and June 2014 in Amsterdam, the Netherlands. A total of 150 patients referred to an independent primary center for colonoscopy (Procolo Amsterdam) were invited to participate. Reasons for referral consisted of rectal blood loss, change in bowel habits, surveillance after polypectomy or CRC, a familial risk of CRC or a positive fecal immunochemical test.

Validity was evaluated in two phases. The first validation phase was conducted between July 2013 and October 2013. Fifty patients participated. Excluded were those not having access to email or internet, unable to speak or read Dutch, and those below 18. Eligible patients received a telephone call in which we explained the purpose of the study and invited them to participate. A personal login code was then provided and a link to the online version of the questionnaire was sent per email.

All study participants were scheduled for an appointment with a registered genetic counselor, who had fulfilled a nationally accredited training in genetic counseling. Participants were asked to fill in the questionnaire before this appointment, which took place an hour before the scheduled colonoscopy. If necessary, one email reminder was sent.

At the appointment the genetic counselor drew a cancer pedigree. This counselor did not have access to the questionnaire responses. Two researchers subsequently decided individually whether a patient had to be referred to a clinical geneticist based on data from the pedigree as well as on questionnaire data by using the before mentioned criteria. Hereafter, for each patient the agreement between referral advice for both family histories was assessed. Only patients with a referral advice based on the pedigree were referred, as this was considered the reference standard.

After the first validation phase, a second validation phase was started. The main objective of this second phase was to evaluate validity of the questionnaire in a larger group. We wanted to evaluate feasibility and performance in subgroups defined by health literacy, nationality, native language and educational level. Health literacy was measured using three validated questions [34], each scored between 0 and 4. Sum scores are divided by 3 and scores higher than 2 were considered to reflect inadequate health literacy [34, 35]. Feasibility was further assessed by measuring the required amount of time needed to complete the questionnaire.

Hundred patients were invited for the second validation phase. In- and exclusion criteria were similar to the criteria in the first validation phase, except for age. To facilitate future introduction of the questionnaire in the national fecal immunochemical test based CRC screening program, an age range of 55–75 years was chosen, similar to the screening program. Patients were invited comparable to the first phase procedure. Reasons for eventual non-participation were recorded.

After completing the questionnaire, a trained researcher repeated all questions in a scripted telephone interview with the participant, several days before the scheduled colonoscopy. After the telephone interview referral decisions were made based on both sets of responses, by two researchers. Discrepancies between questionnaire answers and telephone answers were evaluated. If a discrepancy was found in referral advice, the advice based on the telephone interview was considered the reference standard and therefore only people with a referral indication based on the telephone interview were referred.

We evaluated the reproducibility of all referral decisions based on the questionnaires, the full pedigree data and the telephone interview, in terms of the inter-observer agreement.

### Statistical analysis

We compared referral decisions based on questionnaire data against decisions for the same patients based on full pedigree and telephone interview data. We considered the latter two to be the clinical reference standard, and expressed results as questionnaire sensitivity and specificity, with corresponding 95 % confidence intervals. The questionnaire sensitivity expresses what proportion of persons with a clinical referral based on full pedigree or telephone interview data would have been referred based on questionnaire responses. Specificity expresses the proportion not referred to a genetics specialist or gastroenterologist based on pedigree or telephone interview data, correctly identified as such based on questionnaire data.

Inter-observer agreement in referral decision was expressed in terms of percentage agreement between two researchers and using Cohen’s κ coefficient, defined as the coefficient of agreement corrected for chance. A κ-value <0.20 was regarded as poor agreement; 0.21–0.40 as fair agreement; 0.41–0.60 as moderate agreement; 0.61–0.80 as good agreement and >0.81 as very good agreement. All statistical analyses were performed using SPSS statistics version 21.

### Ethical approval

This study was approved by the institutional review board of the academic medical center in Amsterdam, the Netherlands. The introduction of the questionnaire was considered a part of standard health care; additional approval or informed consent were not required according to Dutch law. Use of the online questionnaire was considered safe as all answers were collected anonymously and could only be linked to a patient with a secured key document. This study was carried out in accordance with the Helsinki Declaration [36].

## Results

### First validation phase

Mean age of the 50 participants in the first validation phase was 57 years (standard deviation 11, range 35–78); 32 (64 %) were female. According to the pedigree data, nine patients had a suspicion of Lynch syndrome, one patient fulfilled the criteria of familial CRC and no persons fulfilled the criteria for a single colonoscopy.

The sensitivity of the questionnaire was 90 % (95 % CI 55–98 %), for a specificity of 98 % (95 % CI 87–100 %) when compared to the pedigree (Table 4). All patients qualifying for referral according to the pedigree data were also detected through the questionnaire, except for one patient with suspected Lynch syndrome. In the pedigree of this patient, for an unknown reason, no age was mentioned for two second degree relatives with a LSAT, whereas the patient had filled out a cancer diagnosis age ‘>70’ in the questionnaire. As we considered an unknown or not reported age as an age ‘50–70’ this resulted in a referral, based on the pedigree data, but not on the questionnaire data.

**Table 4:** Referrals in the first validation phase with 50 participants

Referral indication	Questionnaire (n)	Pedigree (n)	False-negative (n)	False-positive (n)	Sensitivity (%, 95 % CI)	Specificity (%, 95 % CI)
Lynch syndrome	9	9	1	1	90 (55–98)	98 (87–100)
Familial colorectal cancer	1	1	0	0		
Single colonoscopy	0	0	0	0		

In contrast, one patient who did not qualify for referral based on the pedigree data did have a referral indication based on the questionnaire. This patient had indicated in the questionnaire that a first degree relative had cervical cancer as well as endometrial cancer, whereas in the pedigree only cervical cancer was noted.

In 47 patients separate questions were answered differently in the questionnaire, compared to the pedigree answers. The most common discrepancy was found in answers to the questions how many second degree relatives were affected with CRC or with other cancer types. In many cases the answer to the questionnaire indicated that the number of affected relatives was ‘unknown’, whereas in the pedigree matching relatives were marked as ‘not affected’. This did not change referral decisions, as we considered ‘unknown’ equivalent to zero persons affected. The second most common discrepancy was found in the observation that participants reported different numbers of affected second degree relatives in the questionnaire compared to pedigree data (for instance 1 vs. 2 affected second degree relatives in respectively the questionnaire and pedigree of a single patient). In most cases this consisted of cancer types other than CRC or LSAT and this did not change referral indications. Discrepancies were also seen in age of cancer diagnosis, cancer type, existence of a hereditary cancer syndrome, having undergone previous genetic tests and having polyps. This did not change referral indications except for the two before mentioned situations.

An inter-observer agreement of 100 % was achieved for referral decisions performed by the two researchers, for the questionnaire as well as for the pedigree data, leading to a Cohen’s κ of 1.

After this first validation phase, small adaptations were made. We added a question to find out if an affected grandparent was from paternal or maternal lineage. A question about the existence of more than ten polyps was removed, as patients found it difficult to estimate the exact number of detected polyps.

### Second validation phase

Between March 2014 and June 2014 the second validation phase was performed. Twenty-two eligible patients declined the invitation to participate; one patient did not want to participate as she was already diagnosed with familial CRC before inclusion, one patient had a family conflict, one patient was dyslectic, one patient did not participate for an unknown reason, and 18 patients did not have the ability to get online. Enrollment was continued until 100 patients were included.

Median age of participants was 66 years (interquartile range 60–71, range 55–75); 44 (44 %) were female, 93 (93 %) were Dutch and the native language was Dutch for 95 (95 %). Health literacy was considered adequate in all participants and educational level was low in most of them (43 %). Median duration to complete the questionnaire was 7 min (interquartile range 5–10, range 3–20). The mean number of answered questions was 27 (standard deviation 5, range 15–41). More details can be found in Table 5.

**Table 5:** Characteristics of participants in the second validation phase

Participants, n	100
Median age, years (interquartile range, range)	66 (60–71, 55–75)
Gender, n (%)	
Female	44 (44)
Male	56 (56)
Health literacy	
Adequate, n (%)	100 (100)
Inadequate, n (%)	0 (0)
Median (interquartile range, range)	0 (0.00–0.58, 0.00–2.00)
Nationality, n (%)	
Dutch	93 (93)
Other	7 (7)
Native language, n (%)	
Dutch	95 (95)
Other	5 (5)
Educational level, n (%)	
Low	43 (43)
Intermediate	27 (27)
High	29 (29)

The sensitivity of questionnaire-based referral decisions was 100 % (95 % CI 63–100 %) at a specificity of 97 % (95 % CI 91–99 %) (Table 6). According to the questionnaire data, eight patients had a Lynch syndrome suspicion, two patients had familial CRC and one patient had an indication for a single colonoscopy.

**Table 6:** Referrals in the second validation phase with 100 participants

Referral indication	Questionnaire (n)	Phone interview (n)	False-negative (n)	False-positive (n)	Sensitivity (%, 95 % CI)	Specificity (%, 95 % CI)
Lynch syndrome	8	5	0	3	100 (63–100)	97 (91–99)
Familial colorectal cancer	2	2	0	0		
Single colonoscopy	1	1	0	0		

After the telephone verification, it became clear that three of eight Lynch syndrome suspected patients did not have a Lynch syndrome suspicion. Referral decisions for familial CRC and single colonoscopies did not change after the telephone interview.

The first false-positive Lynch syndrome referral concerned a participant who had filled out that her mother had CRC and ovarian cancer, at an age between 50 and 70 years. In the telephone interview this patient said that her mother had ovarian cancer that had ‘spread throughout the abdomen’ and existence of CRC was not confirmed.

The second false-positive referral concerned a participant who had indicated in the questionnaire that a second degree relative had been diagnosed with endometrial cancer at an age younger than 50. In the telephone interview he explained that it most likely involved cervical cancer, which changed the referral indication.

The third false-positive referral was explained by a participant who had filled out that she had three second degree relatives with CRC at an unknown age. In the telephone interview she explained that she had unintentionally given incorrect answers, but she had not been able to change those. In two cases patients were referred to a clinical geneticist according to the questionnaire data as well as the telephone data, but the exact reason for referral had changed after the telephone interview.

Forty-six participants provided different answers in the telephone interview, which only led to different referral indications in the three above mentioned cases. Most of the other different answers were related to the number of second degree relatives with any kind of cancer.

An inter-observer agreement of 100 % was achieved for referral indications based on the questionnaire data, performed by two researchers, leading to a Cohen’s κ of 1.

## Discussion

We developed and validated an online questionnaire to document familial cancer history, in order to facilitate the detection of patients with a suspicion of Lynch syndrome, familial CRC and patients with a CRC family history who qualify for a single colonoscopy. In a two-phase validation process we observed that this questionnaire has a high sensitivity and specificity for detecting an indication for referral to a clinical geneticist or gastroenterologist, compared to referral decisions based on pedigree data collected by a genetic counselor. In the second validation phase data were verified in a telephone interview, with similarly high sensitivity and specificity. Risk assessment based on the given answers in the questionnaire showed a very good inter-observer agreement.

Looking into more detail, there was only one false-negative referral, which was probably due to the accidental non-reporting of the ages of affected family members in the pedigree. False-positive referrals were mainly due to participants’ unawareness of the difference between endometrial or cervical cancer, which we tried to remedy by adding a textual explanation of cervical cancer in the final questionnaire.

As expected, the proportion of patients that had to be referred to a clinical geneticist or gastroenterologist in our study group was higher than the proportion of patients in the general population assumed to have an increased risk of familial or hereditary CRC: 12 % (18/150) versus 2–4 % [5, 37]. This could be explained by the fact that our study group included patients referred for colonoscopy, including referrals for a family history of CRC. As such, this could have also affected the performance of the tool. Unfortunately, outcomes after referral to a clinical geneticist could not be included in this study report, as not all genetic results were available at the time of our analyses.

Several tools have been previously proposed to document family history and to increase the detection of patients with a risk of familial or hereditary CRC, ranging from paper or digital questionnaires to structured interviews [10, 25–29, 38, 39]. However, most of these tools did not take familial CRC into account and a full family history was not documented. A tool that documents the family history and which can also be used to calculate the risk of several CRC syndromes is therefore preferable.

We believe online questionnaires are to be preferred over paper questionnaires as they are easier and less expensive to use and can be used on a large scale [25, 38, 40, 41]. In several studies self-administered answers in online questionnaires were shown not to differ from answers to paper questionnaires [42, 43]. Another benefit of a self-administered online tool is the possibility to fill in the questionnaire at home at any preferred time and thereby saving consulting time at a physician. In case an answer is unknown, the participant can easily contact a family member to collect reliable data, which could potentially increase sensitivity [41]. The structured way of asking questions about relatives, using conditional questions, cannot be efficiently done in a paper questionnaire as this would result in a long and unclear instrument.

Most studies that validated online tools for collecting family history details with a focus on detecting familial CRC and Lynch syndrome did not compare the family history with a reference standard [25, 28, 29, 39]. In the first validation phase we used a pedigree drawn by an experienced genetic counselor for the comparison of the collected family history. In a recent Australian study a sensitivity of 76 % and a specificity of 92 % were reported for detecting patients with a familial risk of CRC when a paper based tool, with self-administered answers, was compared against pedigree data [29]. These estimates are lower than in our study and could possibly be explained by the fact that they did not systemically query the family history and different criteria for an increased familial risk might have been used. In another study self-administered family history details were compared to pre-existent medical chart information [39]. A geneticist compared the results and determined referral indications, but no pedigree was drawn.

In our study we tried to identify persons with Lynch syndrome as well as individuals with familial CRC [11]. This aim differs from that of frequently used digital tools that only identify persons at a risk for Lynch syndrome, such as the PREMM_1,2,6_ and MMRpro models [10]. A recent Dutch study used criteria similar to ours and focused on Lynch syndrome as well as familial CRC [25]. It showed a sensitivity of 91 % for their online referral test, but this was only based on detecting Lynch syndrome mutation carriers in CRC patients. Sensitivity was 73 % for detecting all affected and non-affected mutation carriers. Specificity was not assessed and no comparison was made with a tailor-made pedigree. Sensitivity and specificity in detecting familial CRC were not reported. Compared to their tool more extra-colonic LSAT were included in our referral criteria and the presence of such tumors at an age above 50 was also taken into account, which possibly increased sensitivity of our tool.

We are also aware of study limitations, most of which involve the accuracy of the family history collection. We are aware that we did not perform a test–retest evaluation as part of the validation process, but it is likely that such an evaluation would be influenced by the conversation with a genetic counselor and by the telephone verification.

We found a proportion of (sub-)questions that were answered differently at the pedigree or at the telephone verification. This would possibly also occur in case patients had two pedigrees drawn within a short time period and is therefore difficult to avoid in clinical practice [44]. In our study the differently answered questions did not change referral indications in most cases.

Additionally, the questionnaire responses in the second validation phase were not compared to a pedigree drawn by a genetic counselor, but to responses in a scripted telephone interview instead and the performance of the trained researcher was not compared to that of a genetic counselor. In several other studies a similar comparison was made with data from medical charts or data collected by trained researchers [6, 45]. We believe that in clinical practice a family history can and probably should be documented by any physician, and not exclusively by genetic specialists despite the known shortcomings [19, 20]. As our questionnaire systematically queries all first and second degree relatives and all cancer types, we think that any physician should be capable of collecting a complete family history using this questionnaire.

Another difficulty with family history collection is the fact that family history was quite often unknown regarding certain relatives. This mostly involved second degree relatives, which is a known problem [41, 44]. As it is impossible to verify all unknown information, we consider this an inevitable issue. We advised participants to contact relatives in case of uncertainties, which is stimulated by allowing people to pause the completion of the questionnaire. Therefore, we believe that we created a way to achieve optimal circumstances for complete information collection.

Even after complete information collection, not all patients at risk will be identified, since the current referral criteria do not detect all patients with a hereditary CRC syndrome [25]. The aim of our study was to validate the accuracy of an online self-administered family history tool, not the referral criteria themselves.

Several limitations regarding the studied participants can be mentioned too. In our study group health literacy was considered sufficient in all participants; most people were native Dutch speakers, educational levels varied and in the second validation phase there was an age restriction. We do not yet know how well this questionnaire will perform in non-native speakers and in people with more limited health literacy. As no age restriction was used in the first validation phase, we believe this questionnaire can also be used in age categories other than 55–75 years. Additionally, we selected participants that were referred to a center for colonoscopy and therefore it is questionable if the questionnaire can be used for non-referred persons. As indications for referral varied amongst participants this selection bias seems limited. And unfortunately, several people were not able to participate due to limited access to internet. Help from a family member or friend could avoid this problem. And as the population is increasingly getting familiar with computers and internet we think this problem will eventually be minimized.

Our questionnaire could be embedded in several settings, ranging from primary care clinics, outpatient clinics and population screening. As the second validation phase was conducted in a population that could represent the population participating in the nationwide fecal immunochemical test based CRC screening program, it could be used for that purpose. To reach a wider population, the test should be validated in different languages and in patients with different degrees of health literacy. If used as a screening tool, answers do not necessarily have to be verified since a sensitivity and specificity that are not perfectly accurate are usually accepted in screening circumstances. However, in individual cases, such as for CRC patients, answers need to be verified. An automatic risk assessment based on the answers can help to determine referral indications and this is currently being developed by our research group. The most efficient way of using this tool needs to be further determined.

In conclusion, this online questionnaire for the detection of persons at risk of familial or hereditary CRC syndromes seems to be an accurate tool that can be easily implemented in several health care situations in order to increase the detection of patients with familial CRC and Lynch syndrome. This will eventually result in appropriate treatment and surveillance recommendations for many persons at risk.
